# Molecular Detection of Tick-Borne Bacteria from *Amblyomma* (Acari: Ixodidae) Ticks Collected from Reptiles in South Africa

**DOI:** 10.3390/microorganisms10101923

**Published:** 2022-09-28

**Authors:** Lehlohonolo S. Mofokeng, Nico J. Smit, Courtney A. Cook

**Affiliations:** Water Research Group, Unit for Environmental Sciences and Management, North-West University, Potchefstroom 2531, South Africa

**Keywords:** *Amblyomma*, endosymbionts, reptiles, South Africa, tick-borne pathogens

## Abstract

Reptiles are hosts for various tick species and tick-associated organisms, many of which are zoonotic. However, little is known about the presence and diversity of tick-borne bacteria infecting reptiles and their ticks in South Africa. *Amblyomma* ticks (*n* = 253) collected from reptiles were screened for the presence of *Coxiella, Anaplasma*, *Rickettsia*, and *Borrelia* species by amplification, sequencing and phylogenetic analysis of the 16S rRNA, 23S rRNA, *gltA*, *OmpA*, and *Flagellin* genes, respectively. This study recorded the presence of reptile associated *Borrelia* species and *Coxiella*-like endosymbiont in South Africa for the first time. Furthermore, a spotted fever group *Rickettsia* species was observed in 7 *Amblyomma marmoreum* and 14 *Amblyomma sylvaticum* from tortoises of genera *Kinixys* and *Chersina*. *Francisella*-like endosymbiont was observed from 2 *Amblyomma latum* collected from the Mozambique spitting cobra, *Naja mossambica*. *Coxiella burnetii* and *Anaplasma* spp., were not detected from the current samples. Although the direct evidence that reptiles can act as reservoir hosts remains to be determined, observations from this study provide indications that reptilian ticks may play a role in the transmission of pathogenic bacteria to homothermic animals. Furthermore, the absence of *Anaplasma* spp., and *C*. *burnetii* does not mean that these pathogens should be completely neglected.

## 1. Introduction

Tick-borne pathogens (TBPs) are responsible for some of the most severe emerging infectious diseases across the world [1,2]. The wildlife-domestic-human interaction has the potential to accelerate the spread of zoonotic tick-borne pathogens that cause significant diseases and eventually death [2,3]. To date, roughly 61% of human diseases have been documented to have a zoonotic origin, and wildlife is associated with approximately 75% of emerging zoonotic infections globally [4]. In South Africa, four tick-borne spotted fever groups (SFG) *Rickettsia* spp. (*Rickettsia africae*, *Rickettsia conorii conorii*, *Rickettsia aeschlimannii* and *Rickettsia sibirica mongolitimonae*) have been associated with human diseases [5,6,7]. Additionally, members of the family Ixodidae are involved in the epidemiology of other pathogens such as *Borrelia*, *Anaplasma* and *Coxiella* spp. An increasing number of studies have implicated reptiles and their associated ticks as potential reservoirs involved in the transmission cycles of TBPs [2,8,9]. This fact is of great concern in tick-borne disease control strategies, as these ectotherms are usually asymptomatic carriers that may serve as reservoirs of the infection. Although diverse tick-borne pathogens are emerging across the world, with documented effects on the health of humans and livestock, studies into their diversity and specific interactions with ticks and their reptilian host species in South Africa are lacking. In particular, the role of these ectotherms in the epidemiology of TBPs remains unknown, despite the fact that exported ticks and reptiles (*Amblyomma marmoreum*, tortoises and lizards) have been implicated with TBPs of livestock [2,10]. This leads, therefore, to a lack of knowledge on the diversity of these pathogens in reptiles and whether or not reptiles can act as reservoir hosts for TBPs infecting domestic animals and humans. 

In addition to pathogens, ticks also harbor various microbiomes which include symbiotic and commensal bacteria [11]. Tick endosymbionts have gained interest over the last decade and are now being investigated due to their physiological significance in ticks [12]. These endosymbionts are involved in the reproduction and growth of ticks by providing these arthropods with the necessary compounds that are absent in their haematophagous diet [13,14]. Furthermore, they are also involved in the maintenance of pathogens in arthropods [15]. To date, three genera of symbionts (*Coxiella*-like endosymbionts, *Francisella*-like endosymbionts, and *Midichloria*) have been documented to be exclusive to ticks, and some of these symbionts are mutualistic, whereas others may be facultative [11,16]. 

Despite the above, the presence and diversity of bacteria associated with ticks of reptiles, particularly those species that can also infest livestock and are close to human settlements, have not been studied in South Africa. This lack of information on tick-borne bacteria of reptilian ticks in South Africa warrants novel research in this area. Furthermore, since the literature related to tick-borne bacteria in reptiles and their associated ectoparasites is very recent, and most of these studies have been conducted in South America [9,17,18], the aim of this study was to investigate the presence of bacterial microorganisms from reptile associated ticks in South Africa.

## 2. Materials and Methods

### 2.1. Sample Collection

Ticks from 36 reptiles were collected in the Ndumo Game Reserve, KwaZulu-Natal (KZN) (32°18′49″ E; 26°54′33″ S) (2014–2022) and the region near the village of Paternoster, Western Cape (WC) (17°51′56″ E; 32°49′2″ S) (2011). Upon collection, the ticks were placed in falcon tubes with a damp piece of cottonwool, kept in a dark, cool environment, and allowed to digest their blood meals for approximately 10–20 days [19,20]. Thereafter, they were fixed in molecular grade 70% ethanol (Sigma-Aldrich, St. Louis, MI, USA). Reptiles were restrained by hand whilst ticks were collected in situ. Sampling was carried out under the relevant permits (KZN: OP 839/2014, OP 4374/2015, OP 4092/2016, OP 4264/2017; WC: 0035-AAA004-00383) and was approved by the North- West University Anim-Care Animal Research Ethics Committee, South Africa (NWU-00372-16-A5). Species identification of the collected ticks was carried out under a Nikon AZ100M stereo-microscope, and guidelines for tick identification were conducted as provided by Theiler [21] and Theiler and Salisbury [22]. The molecular methods as described in [20] were used to confirm morphological identification. 

### 2.2. PCR and Phylogenetic Analysis

The DNA from whole tick specimens were extracted using the NucleoSpin^®^Tissue Genomic DNA Tissue Kit (Machery-Nagel, Duren, Germany). DNA was then frozen at −20 °C for further molecular studies.

Molecular characterization of bacterial microorganisms was performed via PCR amplification, amplifying different bacterial genes (Table 1). Conditions for both PCR and nested PCR of all bacteria were as follows: initial denaturation at 95 °C for 3 min, followed by 35 cycles, entailing a 95 °C denaturation for 30 s, annealing at different temperatures as indicated in Table 1, for 30 s with an end extension at 72 °C for 2 min, and following the cycles a final extension of 72 °C for 10 min. PCR reactions were conducted in a 25 μL reaction mixture containing 12.5 μL Thermo Scientific DreamTaq PCR master mix (2×) (2× DreamTaq buffer, 0.4 mM of each dNTP, and 4 mM MgCl_2_), 1.25 μL of each primer (10 μM), and at least 25 ng of DNA template. PCR grade nuclease free water (Thermo Scientific, Vilnius, Lithuania) was used to make up the final reaction volume. Reactions were undertaken in a Bio-Rad C1000 Touch™ Thermal Cycler PCR machine (Bio-Rad, Hemel Hempstead, UK). PCR and nested PCR amplicons were visualized on 1% GelRed^®^ (Biotium Inc., Hayward, CA, USA) stained agarose gels. Visualization was performed under UV light. 

The positive products of PCR and nested PCR were purified and sequenced by Inqaba Biotechnical Industries (Pty) Ltd. (Pretoria, South Africa). The obtained sequences were compared with GenBank references using the Basic Local Alignment Search Tool (BLAST), and deposited in the NCBI GenBank database. 

In addition, phylogenetic reconstruction was performed. For phylogenetic analysis, comparative sequences of the *Coxiella* spp., *Borrelia* and *Rickettsia* species were downloaded from GenBank and aligned to the sequences generated within this study. Sequences were aligned using the Clustal W alignment tool under the default settings and implemented in MEGA Ver 11.1 [23]. To infer phylogenetic relationships, the maximum likelihood (ML) method was used. Prior to the analyses, a model test was performed to determine the most suitable nucleotide substitution model according to the Akaike information criterion (AIC) using jModelTest 2.1.7 [24,25]. For the ML analysis, nodal support was assessed using 1000 rapid bootstrap inferences.

**Table 1:** List of primer sequences used for the detection of bacterial species.

Species	Assay	Primer Sequence	Annealing (°C)	Product Size	Target Gene	References
Universal bacterial primers	PCR	TCAAAKGAATTGACGGGGGC GCCCGGGAACGTATTCAC	50	478 bp	16S rRNA	[26]
*Anaplasma* spp.	PCR	CAGAGTTTGATCCTGGCTCAGAACG GAGTTTGCCGGGACTTCTTCTGAA	50	467 bp	16S rRNA	[27]
*Borrelia* spp.	PCR	AGAGCAACTTACAGACGAAATTAAT CAAGTCTATTTTGGAAAGCACCTAA	54	482 bp	*Flagellin* gene	[28]
*Borrelia* spp.	PCR	TGCGTCTTAAGCATGCAAGT GTACAAAGGCCCGAGAACGTA	56	1344 bp	16S rRNA	[29]
	nPCR	CATGCAAGTCAAACGGAATG ACTGTTTCGCTTCGCTTTGT	56	1205 bp	16S rRNA	[29]
*Rickettsia* spp.	PCR	GGGGGCCTGCTCACGGCGG ATTGCAAAAAGTACAGTGAAC	48	382 bp	*gltA* gene	[30]
*Rickettsia* spp.	PCR	TGGCGAATATT TCTCCAAAAA GTTCCGTTA ATGGCAGCATCT	46	631 bp	*ompA* gene	[31]
*Coxiella* spp.	PCR	CGTAGGAATCTACCTTRTAGWGG GCCTACCCGCTTCTGGTACAATT	52	832–939 bp	16S rRNA	[32]
	nPCR	CGTAGGAATCTACCTTRTAGWGG TGAGAACTAGCTGTTGGTTAGT	52	624–627 bp	16S rRNA	[32]
*Coxiella* spp.	PCR	GCCTGCGAWAAGCTTCGGGGAG CTCCTAKCCACASCTCATCCCC	56	694–1188 bp	23S rRNA	[32]
	nPCR	GATCCGGAGATWTCYGAATGGGG TCGYTCGGTTTCGGGTCKACTC	56	583–867 bp	23S rRNA	[32]
*Coxiella burnetii*	PCR	ACT CAA CGC ACT GGA ACC GC TAG CTG AAG CCA ATT CGC C	50	257 bp	*htpAB*	[33]

### 2.3. Statistical Analysis

The 95% confidence interval was calculated using the formula: P ± Z × $\frac{\sqrt{P\;\left(1-P\right)}}{n}$.

## 3. Results

### 3.1. Overall Prevalence

Two hundred and fifty-three ticks (80 males, 21 females, and 12 larval stages of *A. marmoreum*; 40 males and three females of *A. latum*; 36 males and one female of *A. exornatum*; and 49 males and 11 females of *A. sylvaticum*) (Table S1) were collected from 36 reptiles and screened for tick-borne bacterial microorganisms (142 ticks in KZN; 111 ticks in WC). Based on the PCR results, all four tick species showed evidence of infection with at least one bacterial microorganism (see Table 2). A spotted fever group *Rickettsia* species was detected from 21 out of 253 samples (8.3%) (95%CI = ±3.39), reptile associated *Borrelia* species was detected in 16 out of 253 samples (6.3%) (95%CI = ±2.99), *Francisella*-like endosymbiont in two out of 253 samples (0.8%) (95%CI = ±1.10), and *Coxiella*-like endosymbiont (CLE) in 104 out of 253 samples (41.1%) (95%CI = ±6.06) (Table 2). Mixed infections of CLE and *Borrelia* spp. were observed in nine out of 253 samples (3.6%) (95%CI = ±2.21). The *Anaplasma* species and *Coxiella burnetii* were not found in the ticks examined.

In KZN, three genera of bacteria were observed in *Amblyomma* ticks. *Borrelia* spp. was observed at a prevalence of 5.6% (95%CI = ±3.78), *Francisella*-like endosymbiont at 1.4% (95%CI = ±1.93), and *Coxiella*-like endosymbiont at 21.1% (95%CI = ±6.71), while in WC, *Borrelia* sp. was observed at 7.2% (95%CI = ±4.81), *Coxiella*-like endosymbiont at 66.7% (95%CI = ±8.77), and *Rickettsia* sp., at 18.9% (95%CI = ±7.28). 

*Francisella*-like endosymbiont was observed only in *A. latum* at a prevalence of 4.7% (95%CI = ±6.33), while *A. sylvaticum* harbored *Rickettsia* sp. at a prevalence of 23.3% (95%CI = ±10.61), *Coxiella*-like endosymbiont at a prevalence of 45.9% (95%CI = ±12.61), and *Borrelia* sp. at a prevalence of 6.7% (95%CI = ±6.33). Three bacterial genera were observed in *A. marmoreum*. *Borrelia* sp. was observed at a prevalence of 10.6% (95%CI = ±5.68), *Coxiella*-like endosymbiont at 35.4%, (95%CI = ±8.82) and *Rickettsia* spp. at 6.2% (95%CI = ±1.16). *Coxiella*-like endosymbiont was observed at a prevalence of 32.4% (95%CI = ±15.08) in *A. exornatum*. Mixed infections of *Coxiella*-like endosymbiont and *Borrelia* sp. were observed at a prevalence of 6.19% (95%CI = ±4.44) in *A. marmoreum*, and 3.33% (95%CI = ±4.52) in *A. sylvaticum.*

### 3.2. Phylogenetic Analysis of Bacterial Microorganisms in Amblyomma Ticks

According to the BLASTn analysis, the ~478 bp fragment of 16S rRNA for the universal bacterial microorganisms of *A. latum* yielded 98–99% with uncultured *Francisella* species (MN998636-MN998638) from France, and these sequences further clustered within the monophyletic clade of previously described FLEs rather than with pathogenic *F. tularensis* (Figure S1). The same gene from *A. sylvaticum* yielded (449/457, 98.25%) with uncultured *Rickettsia* spp. isolated from *Amblyomma helvolum* and *Aponomma varanensis* (KF318168.1 and JQ412124.1) in Thailand, and this sequence was submitted to GenBank as uncultured *Rickettsia* sp. under accession number (OP068356).

**Figure 1:**
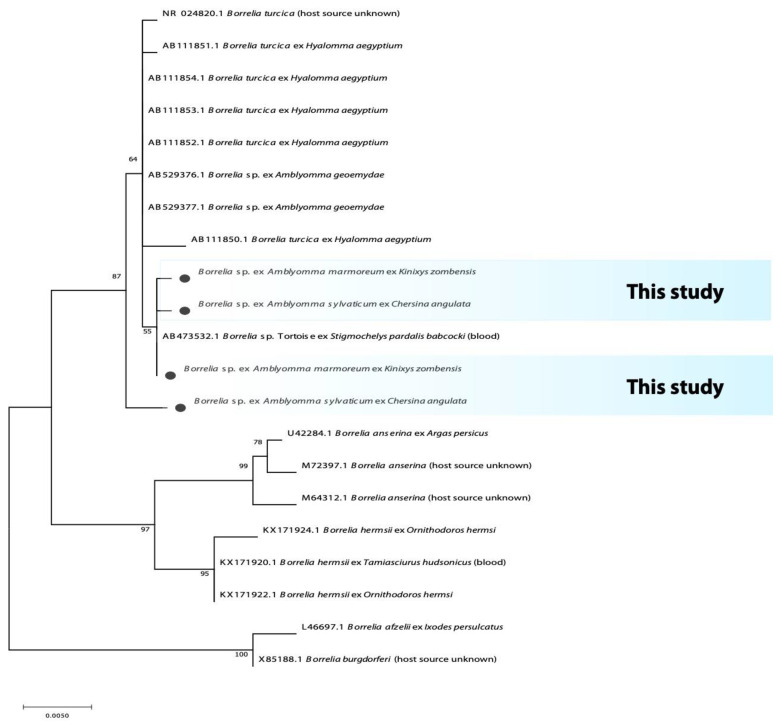
Phylogenetic tree based on the sequences of the *Borrelia* 16S rRNA gene. The tree was constructed using MEGA 11 based on the maximum likelihood, using the Hasegawa-Kishino-Yano Model. The sequences obtained in this study are indicated with bullets.

**Figure 2:**
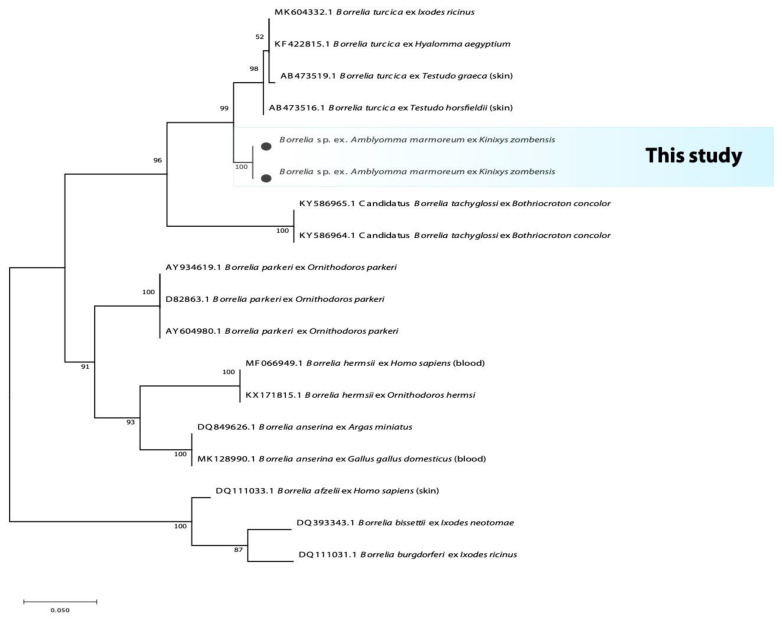
Phylogenetic tree based on the sequences of the *Borrelia Flagellin* gene. The tree was constructed using MEGA 11 based on the maximum likelihood, using the Tamura 3-parameter Model. The sequences obtained in this study are indicated with bullets.

The 482 bp fragment of the flagellin gene (Fla) from *A. marmoreum* yielded an identity of 95% with *Borrelia turcica* (MK604332.1) from Poland, while the 1205 bp fragment of the 16S rRNA from both *A. marmoreum* and *A. sylvaticum* yielded an identity of 99.3% with *Borrelia* sp. tortoise (AB473532.1) and *Borrelia turcica* (CP028884.1). The sequences from this study fall within a monophyletic group with other sequences of the reptile-associated *Borrelia* group that is distinct but close with relapsing fever borreliae (Figure 1 and Figure 2). 

**Figure 3:**
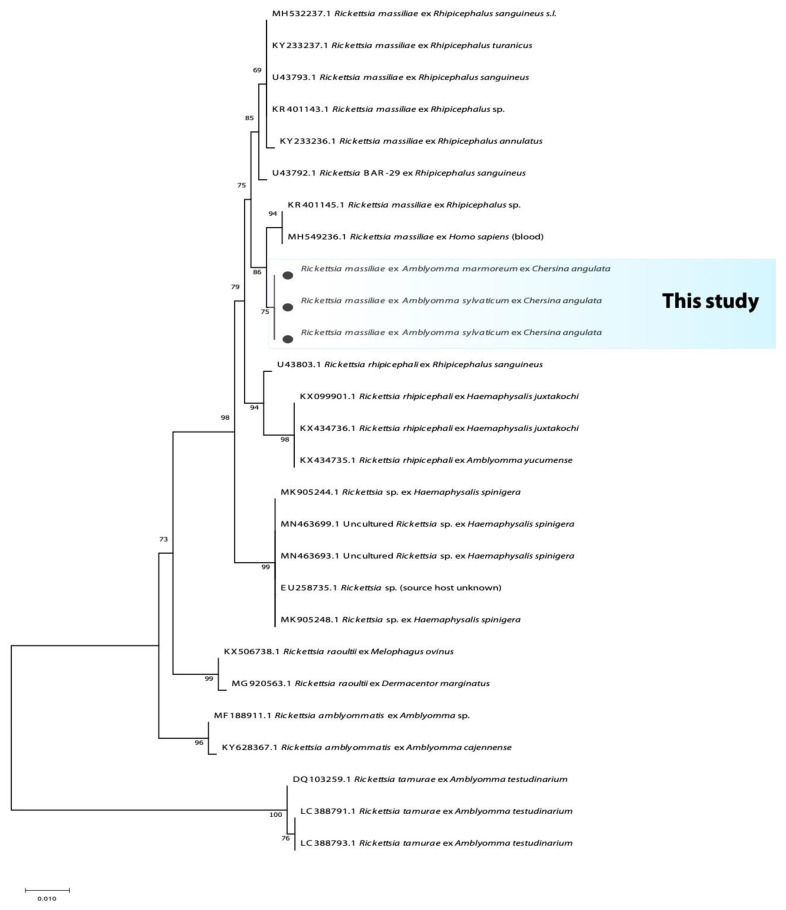
Phylogenetic tree based on the sequences of the *Rickettsia OmpA* gene. The tree was constructed using MEGA 11 based on the maximum likelihood, using the Tamura 3-parameter model. The sequences obtained in this study are indicated with bullets.

On the other hand, the 396 bp fragment of the citrate synthase gene (*gltA*) for *Rickettsia* amplified from *A. marmoreum* and *A. sylvaticum* yielded an identity of 97.9% with the spotted fever group *Rickettsia massiliae* (KY640405.1) and *R. rhipicephali* (KX018048.1). However, all the sequences of the 631 bp fragment of ompA gene for *Rickettsia* amplified from both *A. marmoreum* and *A. sylvaticum* yielded 99.1% with only *R. massiliae* (MH549236.1 and KR401145.1). The phylogenetic analysis of the fragment of the *ompA* clusters the current study’s *Rickettsia* sequences recovered from *Amblyomma* ticks in a monophyletic group with a support value of 75%. With high support (86%), these sequences form a sister clade to *R. massiliae*; this group in turn clusters within a larger monophyletic clade containing unnamed *Rickettsia* species and *R. massiliae* (Figure 3). 

**Figure 4:**
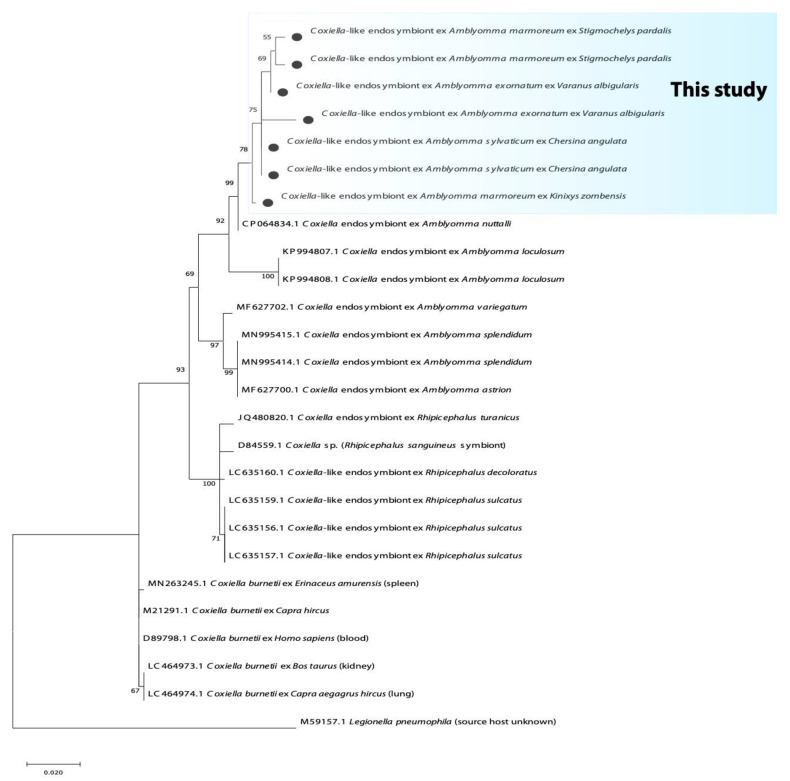
Phylogenetic tree based on the sequences of the *Coxiella* 16S rRNA gene. The tree was constructed using MEGA 11 based on the maximum likelihood, using the Kimura 2-parameter model. The sequences obtained in this study are indicated with bullets.

**Figure 5:**
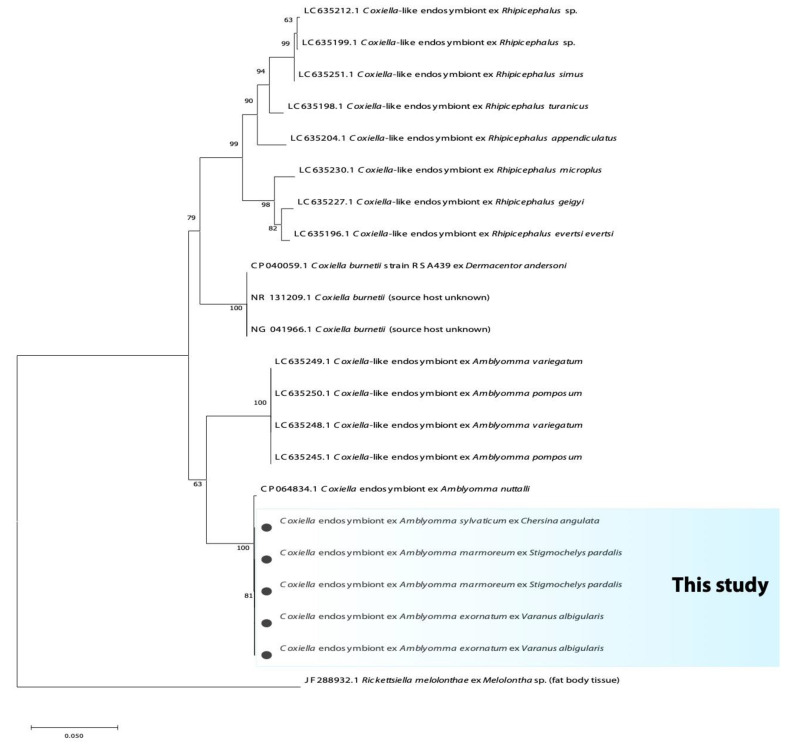
Phylogenetic tree based on the sequences of the *Coxiella* 23S rRNA gene. The tree was constructed using MEGA 11 based on the maximum likelihood, using the Kimura 2-parameter model. The sequences obtained in this study are indicated with bullets.

The fragment of the 16S rRNA and 23S rRNA yielded an identity of 99% with the complete genome of *Coxiella*-like endosymbiont (CP064834.1) from *Amblyomma nutalli*. Furthermore, these sequences also yielded an identity of 95.9% with the complete genome of the *Coxiella burnetii* strain RSA439 (CP040059.1) and *C. burnetti* isolate from *Hyalomma asiaticum* (MN880312.1). The phylogenetic analysis based on the 16S rRNA gene and 23S rRNA showed that *Coxiella*-like endosymbiont from reptilian *Amblyomma* ticks are grouped into an independent and distinct clade; it further clustered in a large monophyletic clade of *Coxiella*-like endosymbionts of *Amblyomma* tick species previously reported (Figure 4 and Figure 5).

The sequences derived during this study were submitted to the GenBank database under the accession numbers: *Coxiella* spp. (16S rRNA gene: OP070964-OP070972; 23S rRNA gene: OP071239-071243); *Borrelia* spp. (16S rRNA gene: OP081024-OP081026; *flagellin* gene: OP082442-OP082443); *Francisella* spp. (16S rRNA: OP068354 & OP068355); *Rickettsia* spp. (16S rRNA: OP068356; ompA gene: OP329173-OP329175).

## 4. Discussion

Notwithstanding the fact that tick-borne bacterial microorganisms represent serious emerging and re-emerging health problems to humans and other vertebrates, the surveillance of these pathogens in wildlife has focused mainly on mammals, birds and their ectoparasites, whereas reptiles and their associated ectoparasites remain unexplored [16,34]. Reptiles from Africa have been reported to carry *Borrelia* and *Rickettsia* infected ticks [8,17]. However, studies focusing on the detection of bacterial microorganisms in these ectothermic hosts, these hosts’ ability to act as reservoirs of infection or their ability to be refractory to infection [34], and their associated ticks, are only recent and scarce, making their inventory incomplete. This demonstrates the critical need to investigate their potential role in introducing and spreading new tick-borne bacterial diseases in South Africa in order to understand their transmission and zoonotic potential.

Beati et al. [35] isolated *Rickettsia massiliae* for the first time from *Rhipicephalus sanguineus s.l.* collected from canines in France, identifying it as a distinct species within the spotted fever group of rickettsiae. It has been mainly associated with ticks of the genus *Rhipicephalus*. It was then detected from various tick species associated with several vertebrate hosts in Europe, Africa and the Middle East [7,8,36,37]. Even though a human case of *R. massiliae* has not been reported in South Africa, it has been reported in Europe and Argentina, and as such should be considered in patients with tick bites [38,39,40]. Recently, this *Rickettsia* species was also detected in *Rhipicephalus* ticks infesting domestic small ruminants in Nigeria [41]. The finding of *R. massiliae* in reptilian ticks from South Africa suggests that human cases of this pathogen can occur. Therefore, ticks associated with reptiles should be considered in the epidemiology of *R. massiliae*, although more studies investigating the potential role of these ticks as vectors and reservoirs of this pathogen are needed. In the current study, *R. massiliae* was detected from *A. sylvaticum* and *A. marmoreum* collected from tortoises in South Africa, and these results are in accordance with a previous report on the presence of *R. massiliae* in *Amblyomma* ticks in this country [8]. However, it is unfortunate that the *R. massiliae* detected by Halajian et al. [8] could not be compared by phylogenetic analysis to *R. massiliae* in the present study, as different genes were amplified between these two studies.

*Anaplasma* species are tick-transmitted intracellular bacteria that infect a multitude of wild and domestic animals, as well as humans [42]. Although *Anaplasma* species have been documented from ticks and reptiles [43,44], they have not been recorded from African reptiles and their associated ticks, and we did not detect *Anaplasma* species in the current samples.

*Coxiella burnetii* is the causative agent of Q fever, a zoonotic disease with a global distribution, and it has been reported throughout the African continent in ticks, animal production and humans [45,46,47,48]. Ticks may become infected with *C. burnetii* during feeding but are not recognized as the actual vectors of the pathogen [49]. However, the current study could not detect *C. burnetii*. Nevertheless, for the first time in South Africa, the present study reports on the occurrence of *Coxiella*-like endosymbiont in reptilian ticks. These endosymbionts have been documented as vitamin provisioning endosymbionts, and it was reported that the biosynthetic pathways for various vitamins are found in all CLE [50,51]. Furthermore, Nardi et al. [14], in their genomic study, have suggested that these CLEs have independently evolved from *C. burnetii.* The prevalence of CLE in the current study was 41.1% among all the screened ticks, and it was detected in three of four *Amblyomma* species identified with different percentages. This was not surprising, as it has been documented that the prevalence of these bacteria varies widely in various species of ticks. This prevalence was in accordance with studies by Arthan et al. [52] and Rahal et al. [53], who reported prevalences of 42.2% and 51.7% in ticks from vegetation and cattle, respectively. Taking this into consideration, the potential of these CLEs to adapt to vertebrate hosts and evolve into pathogenic *Coxiella* cannot be ruled out. Recently, CLE was confirmed to be a new agent of human infections such as neck lymphadenopathy syndrome and scalp eschar through tick bites [54]. Furthermore, Shivaprasad et al. [55] and Vapniarsky et al. [56] reported avian infections of *Coxiella*-like bacteria even though there was no link to ticks. Therefore, the pathogenicity and zoonotic potential of these *Coxiella*-like bacteria merits further investigation. The negative results for *Coxiella*-like bacteria in *A. latum* may be due to the presence of other symbionts such as *Francisella*-like bacteria, as it was detected in some of the *A. latum* using bacteria specific primers, thus the presence of other endosymbionts in these ticks must not be ruled out. 

Although *Anaplasma* spp., and *C. burnetii* were not detected, they cannot be completely disregarded, as their absence could be attributed to the limited number of ticks and localities analysed in the current study. More studies should be done in more localities to assess the impact of these bacterial microorganisms in reptiles and the risk of transmission to domestic animals and humans.

Takano et al. [57] identified reptile-associated-*Borrelia* spp. as a novel group of *Borrelia* spp. from imported reptiles and/or their ectoparasites, although this group have been found to be associated with species of the order Monotremata [29,58,59]. The transmission of these species is thought to be through hard-bodied ticks belonging to the genera *Hyalomma* or *Amblyomma,* while they appear to have evolutionarily diverged from an ancestor shared with RF *Borrelia* spp. [57]. In Africa, these species were only documented from *Amblyomma* ticks. Reptile-associated-*Borrelia* isolated from *Amblyomma* ticks clustered with the RF *Borrelia* based on a phylogenetic analysis study conducted by Takano et al. [60]. However, in 2014, based on the analysis of the 16S rRNA gene, Adelou and Gupta [61] grouped reptilian *Borrelia* species in a third distinct clade.

In this study, *Borrelia* spp. were detected in two species of *Amblyomma* ticks collected from *Chersina angulata* and *Kinixys* spp., and it was closely related to the REP borreliae group. This group has been previously detected in reptiles and their ticks imported from Africa into Japan [57]. These findings showed the presence of REP *Borrelia* in South Africa for the first time. *Amblyomma marmoreum* have been reported to feed on mammals and exported reptiles [62], which causes a serious concern on the spreading of pathogens by these *Amblyomma* ticks. Thus far, the impact of REP borreliae in humans and animals in South Africa is unknown. However, more detailed phylogenetic studies of the four groups of the genus *Borrelia* are needed to elucidate the origin of REP borreliae and that in turn may aid in clarifying the taxonomic status of species in this genus [63,64].

Moreover, co-infection of *Coxiella*-like endosymbiont and *Borrelia* sp. was found in *A. marmoreum* and *A. sylvaticum* collected from tortoises. This could be due to co-transmission of the pathogens during blood feeding on the host, or as a result of infection during different feedings [65,66].

Recently, many species of reptiles have been and continue to be traded and kept as pets in several countries across the world. As a result, tick-borne pathogens pose potential health threats to humans, associated pets and livestock; therefore, knowledge of the dynamics of the circulation of tick-borne bacteria in the environment is important to assess the risk of infection to both livestock and humans. Although the direct evidence remains to be determined, observations from this study provide clues for the possibility that reptile associated *Amblyomma* ticks can play a role in the maintenance and transmission of pathogenic bacteria to homothermic animals. The accumulation of this epidemiological information from multiple individuals and/or species of reptiles and different stages of ticks in future studies should be informative for this goal. More work will need to be done to determine the prevalence and pathogenic potential of *R. massilae*, REP *Borrelia* and *Coxiella*-like endosymbionts in South Africa. Recognition of the potential of *Rickettsia* species, *Borrelia* species and CLEs in reptiles and their *Amblyomma* ticks will help further our understanding of the emerging bacterial diseases in the country and the potential threat they pose to a global market in reptiles and their associated products.

This study also observed *Francisella*-like endosymbionts from *Amblyomma latum* using bacteria-specific primers. Though the role of most bacteria has not yet been clearly elucidated, the essential role of *Francisella*-like endosymbionts have been reported in several tick species in which it may provide essential nutrition for the tick [58,67,68].

## 5. Conclusions

The present study demonstrates for the first time the presence of REP *Borrelia* and *Coxiella*-like endosymbionts in *Amblyomma* ticks infesting South African reptiles, and corroborates the presence of *R. massilae* in South Africa. These results should increase the awareness on the emergence of these pathogens in this region, and additional studies should elucidate the role of these ectotherms in the transmission of different tick-borne bacterial microorganisms to domestic animals and humans. Findings from this study should stimulate further wide-scale investigation to determine the role of *Francisella*-like endosymbionts and *Coxiella*-like endosymbionts in tick physiology and vector competence.

## Figures and Tables

**Table 2:** Prevalence of bacterial microorganisms from reptilian *Amblyomma* ticks.

Province	Tick Species	*n*	Host (*n*)	16S rRNA Bacteria	*Rickettsia* spp.		*Borrelia* spp.		*Coxiella* spp.	
					*gltA* Gene	*OmpA* Gene	*Flagellin* Gene	16S rRNA	16S rRNA	23S rRNA
KZN	*A. marmoreum*	4	*Varanus albigularis* (1)	-	-	-	-	-	50% (2/4)	-
	*A. marmoreum*	13	*Kinixys* sp. (1)	-	-	-	46.2% (6/13)	46.2 (6/13)	46.2% (6/13)	30.8% (4/13)
	*A. marmoreum*	11	*Kinixys zombensis* (1)	-	-	-	18.2% (2/11)	18.2% (2/11)	45.5% (5/11)	45.45% (5/11)
	*A. marmoreum*	12	*Stigmochelys pardalis* (7)	-	-	-	-	-	41.7% (5/12)	16.7% (2/12)
	*A. latum*	43	*Naja mossambica* (3), *Dendroapis* spp. (4)	4.7% (2/43) *Francisella* spp.	-	-	-	-	-	-
	*A. exornatum*	37	*Varanus albigularis* (5)	-	-	-	-	-	32.4% (12/37)	24.3% (9/37)
WC	*A. marmoreum*	21	*Kinixys zombesis* (3)	-	-	-	4.8% (1/21)	4.8% (1/21)	53.4% (11/21)	38.1% (8/21)
	*A. marmoreum*	37	*Stigmochelys. pardalis* (2)	-	-	-	-	-	56.8% (21/37)	40.5% (15/37)
	*A. marmoreum*	15	*Chersina angulata* (9)	-	46.7% (7/15)	33.3% (5/15)	20% (3/15)	20% (3/15)	53.3% (8/15)	53.3% (8/15)
	*A. sylvaticum*	60	*Chersina angulata* (9)	1.7% (1/60) *Rickettsia* spp.	20.0% (12/60)	23.3% (14/60)	-	6.7% (4/60)	56.7% (34/60)	23.3% (14/60)

(-) = negative for specific pathogen; KZN = KwaZulu-Natal; WC = Western Cape; *n* = number of ticks collected. Number of vertebrate hosts collected is indicted in parentheses next to the host’s name.

## Data Availability

The data presented in this study are openly available in the NCBI Databases.

## Supplementary Materials

The following supporting information can be downloaded at: https://www.mdpi.com/article/10.3390/microorganisms10101923/s1, Table S1: Tick bacteria data; Figure S1: Phylogenetic tree based on the sequences of the 16S rRNA gene.
