# Supplementary material for: Molecular Detection of Tick-Borne Bacteria from Amblyomma (Acari: Ixodidae) Ticks Collected from Reptiles in South Africa
Source: Microorganisms. 2022 Sep 28;10(10):1923. doi: 10.3390/microorganisms10101923 (PMC9607068; doi:10.3390/microorganisms10101923)
Supplement: Supplementary file 1 [file microorganisms-10-01923-s001.zip › microorganisms-1924076-supplementary/SUPP/microorganisms-1924076-supplementary.pdf]

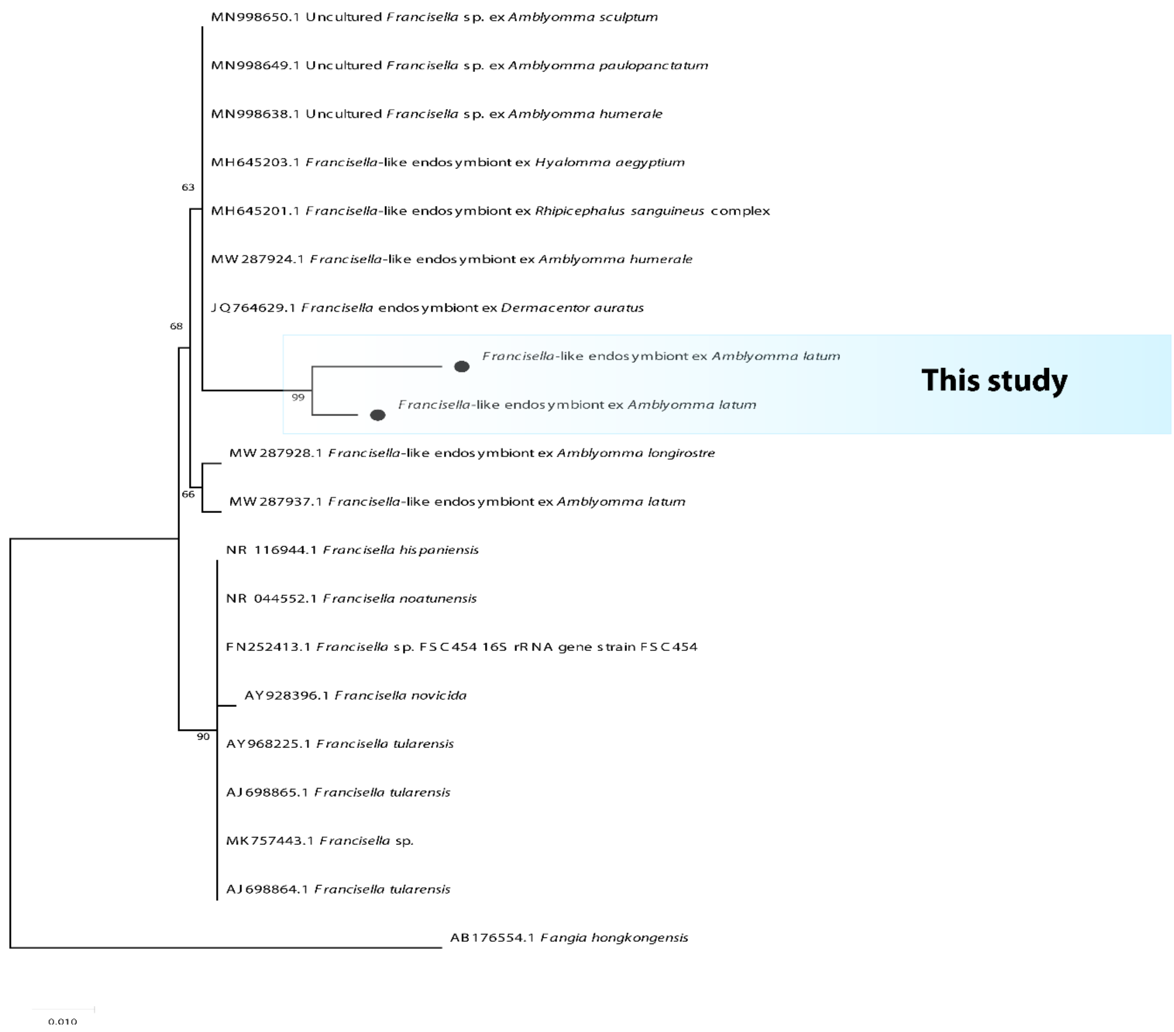

Figure S1. Phylogenetic tree based on the sequences of the *Francisella* 16S rRNA gene. The tree was constructed using MEGA 11 based on the maximum likelihood method, using the Jukes-Cantor Model. The sequences obtained in this study are indicated with bullets.
